# 亚实性肺结节CT征象在良恶性鉴别及腺癌恶性侵袭程度评估中的价值

**DOI:** 10.3779/j.issn.1009-3419.2018.06.05

**Published:** 2018-06-20

**Authors:** 芳芳 郭, 欣菱 李, 欣悦 王, 文松 郑, 卿 王, 文静 宋, 铁链 于, 亚光 范, 颖 王

**Affiliations:** 1 300052 天津，天津医科大学总医院医学影像科 Department of Radiology, Tianjin Medical University General Hospital, Tianjin 300052, China; 2 050011 石家庄，石家庄第一医院影像科 Department of Radiology, Shijiazhuang First Hospital, Shijiazhuang 050011, China; 3 050001 石家庄，河北医科大学第三医院影像科 Department of Radiology, The Third Hospital of Hebei Medical University, Shijiazhuang 050001, China; 4 300052 天津，天津医科大学总医院病理科 Department of Pathology, Tianjin Medical University General Hospital, Tianjin 300052, China; 5 300052 天津，天津肺癌转移与肿瘤微环境重点实验室，天津市肺癌研究所，天津医科大学总医院 Tianjin Key Laboratory of Lung Cancer Metastasis and Tumor Microenvironment, Tianjin Lung Cancer Institute, Tianjin Medical University General Hospital, Tianjin 300052, China

**Keywords:** 肺肿瘤, 亚实性结节, 计算机体层成像, 定量变量, 定性变量, 良性, 恶性, Lung neoplasms, Subsolid nodules, Computed tomography, Quantitative variables, Qualitative variables, Benign, Malignant

## Abstract

**背景与目的:**

亚实性肺结节为肺原发腺癌的常见计算机体层成像（computed tomography, CT）表现，依据其CT影像特征预测病理分型对确定临床治疗策略具有临床价值。本研究根据病理分类，回顾性分析良性、不典型腺瘤样增生（atypical adenomatous hyperplasia, AAH）/原位腺癌（adenocarcinoma *in situ*, AIS）/微侵袭性腺癌（minimally invasive adenocarcinoma, MIA）、侵袭性腺癌三组亚实性肺结节的CT征象，评估其在良恶性鉴别及恶性侵袭程度判别中的价值。

**方法:**

回顾性分析106例经手术切除亚实性结节的CT征象。依据手术病理分为良性和恶性组，恶性组根据侵袭程度分为无/微侵袭组（AAH/AIS/MIA）、侵袭性腺癌组，测量结节大小、实性成分比例、瘤肺界面、形状、边缘、胸膜牵拉征、空气支气管征、结节内血管异常等CT征象。根据单因素分析（χ^2^检验、非参数检验*Mann-Whitney U*检验）结果筛选有统计学差异的变量，纳入*Logistic*回归多因素分析。

**结果:**

*Logistic*回归分析显示清晰的瘤肺界面、空气支气管征以及结节内血管异常是恶性结节的重要预测指标，风险比分别为38.1（95%CI: 5.0-287.7; *P* < 0.01）、7.9（95%CI: 1.3-49.3; *P*=0.03）、7.2（95%CI: 1.4-37.0; *P*=0.02）。更大的实性成分所占比例是侵袭性腺癌与AAH/AIS/MIA组鉴别的重要指标，其风险比分别为1.04（95%CI: 1.01-1.06, *P*=0.01）。

**结论:**

亚实性结节中出现清晰的瘤肺界面、空气支气管征、结节内血管异常提示其恶性概率增加。恶性结节中实性成分所占比例越大预示着侵袭性更高。

## Abstract (en)

**Background and objective:**

Subsolid pulmonary nodules are common computed tomography (CT) findings of primary lung adenocarcinoma. It is of clinical value to determine the clinical treatment strategies based on CT features. The aim of this study is to find the valuable CT characteristics on differential diagnosis and the degree of invasion prediction by a retrospectively analysis of three groups subsolid nodules, including benign, and invasive adenocarcinoma.

**Methods:**

The CT findings of 106 cases of resected sub-solid nodules were retrospectively analyzed. The nodules were firstly divided into benign and malignant groups and the malignant group was further divided into non/micro-invasive group (atypical adenomatous hyperplasia/adenocarcinoma *in situ*/minimally invasive adenocarcinoma) and invasive adenocarcinoma group. The nodule size, proportion of solid components, tumor-lung interface, shape, margin, pleural traction, air bronchus sign, vascular abnormalities inside the nodule were evaluated. The univariate analysis (χ^2^ test, non-parametric test *Mann-Whitney U* test) was performed to screen statistically significant variables and then enrolled in further multivariate *Logistic* regression analysis.

**Results:**

Multivariate logistic regression analysis showed that a clear tumor-lung interface, air bronchus sign, and pulmonary vascular abnormalities were important indicators of malignant nodules with hazard ratios of 38.1 (95%CI: 5.0-287.7; *P* < 0.01), 7.9 (95%CI: 1.3-49.3; *P*=0.03), 7.2 (95%CI: 1.4-37.0; *P*=0.02), respectively. The proportion of solid components was the only significant indicator for identifying invasive adenocarcinoma from AAH/AIS/MIA, with a risk ratio of 1.04 (95%CI: 1.01-1.06, *P*=0.01).

**Conclusion:**

SSNs with clear tumor-lung interface, air bronchus sign, and pulmonary vascular abnormality inside nodule are more likely to be malignant. A higher percentage of solid components indicates a higher likelihood to be an invasive lesion in malignant SPNs.

亚实性肺结节（subsolid nodules, SSNs）包括磨玻璃密度结节（ground-glass nodules, GGNs）和部分实性结节（part-solid nodules, PSNs）。GGNs定义为肺内模糊的、密度稍大于肺组织而未掩盖支气管、血管的结节; PSNs定义为结节内既包含磨玻璃密度成分也包含实性软组织密度成分^[1, 2]^。亚实性结节可能是由感染、炎症、出血或恶性肿瘤引起^[3]^，其恶性概率较实性肺结节高，病理常表现为腺癌。

本研究根据国际肺腺癌最新病理分类，回顾性分析良性、不典型腺瘤样增生（atypical adenomatous hyperplasia, AAH）/原位腺癌（adenocarcinoma *in situ*, AIS）/微侵袭性腺癌（minimally invasive adenocarcinoma, MIA）、侵袭性腺癌三组亚实性肺结节的计算机体层成像（computed tomography, CT）征象，评估其在良恶性鉴别及恶性侵袭程度判别中的价值。

## 1 材料与方法

### 1.1 患者资料

回顾性分析2012年1月-2017年6月在天津医科大学总医院医学影像科行胸部多层螺旋CT（multi-slice spiral CT, MSCT）检查且经手术治疗的SSN病例。入选标准：①有至少一个纯磨玻璃密度结节或部分实性结节; ②有薄层胸部CT图像（层厚=1.25 mm）; ③结节直径 > 5 mm，结节的实性成分 < 3 cm; ④结节手术切除病理为恶性的结节必须为腺癌并且按2011年国际肺癌研究协会（the International Association for the Study of Lung Cancer, IASLC）/美国胸科学会（American Thoracic Society, ATS）/欧洲呼吸学会（European Respiratory Society, ERS）的肺腺癌分类标准分类; ⑤无恶性肿瘤病史。如果病人有多个切除的亚实性结节，且符合以上条件均纳入研究。

本研究入选99例共106个被切除的亚实性结节，其中93例1个结节，5例2个结节，1例3个结节。99例患者中，42例为男性，57例为女性，平均年龄62岁（范围35岁-79岁）。106个结节中12例为良性（4例炎性病变，2例炎性假瘤，3例纤维组织增生，2例错构瘤，1例Wegener肉芽肿），12例为AAH/AIS/MIA（3例AAH，3例AIS，6例MIA），82例为侵袭性腺癌。

### 1.2 图像采集

所有检查由16排（General Electric Company, GE）或64排（Light Speed VCT; Discovery HD CT; Optima）螺旋CT机进行扫描。扫描范围自胸廓入口至肺底部，患者一次吸气后屏气完成全肺扫描。扫描方式：螺旋扫描; 管电压120 kV或140 kV; 管电流：200 mA-340 mA; 螺距：1.375:1;层厚：5.0 mm; 图像矩阵：512×512;视野（field of view, FOV）：360 mm。用标准算法重建1.25 mm层厚轴位图像。

### 1.3 图像分析

所有的CT图像分析在GE AW4.6工作站上完成。有2名影像医师（分别有3年和2年的胸部CT诊断经验）在对病理结果不知情的情况下分析图像。结节CT特征包括定性变量：（1）结节类型（纯磨玻璃密度结节，部分实性结节），实性成分在肺窗上判定; （2）形状（球形/椭球形，不规则形），椭球形结节的最长径/最短径 < 2;（3）瘤肺界面（清晰，不清晰）; （4）边缘（光滑，毛刺，分叶）; （5）空泡征; （6）空气支气管征; （7）胸膜牵拉征; （8）结节内肺血管异常（扩张，僵直或扭曲）; （9）多发（有两个或两个以上亚实性结节）; （10）位置（叶）; （11）肺周分布（肺中、外1/3）。定量变量包括：（1）长径（最大横截面的最长径）、短径（最大横截面最长径的垂直径）、高度（冠状面纵向最长径）; （2）兴趣区平均CT值和它的标准差（standard deviation, SD）; （3）兴趣区的选取：在结节最大横截面上选取最大的圆形兴趣区并尽可能的避免含血管、支气管; （4）结节体积、实性成分体积、实性成分所占百分比、结节平均CT值（使用高级肺结节分析（advanced lung analysis, ALA）软件测得）; （5）质量，质量（g）=（结节平均CT值+1, 000）/10^6^×结节体积（mm^3^）。定量变量使用两位影像医师测的平均值，定性变量由两位影像医师共同商议得到最终结果。

### 1.4 病理

所有组织切片由两名病理学家进行组织病理学分析，最终达成共识。腺癌根据IASLC/ATS/ERS 2011肺腺癌分类标准对样本进行分析（AAH、AIS、MIA和侵袭性腺癌）。

### 1.5 统计学方法

将亚实性结节按病理首先分为良性组和恶性组，恶性组再根据恶性程度及预后分为AAH/AIS/MIA组、侵袭性腺癌组。尽管MIA是侵袭性腺癌，但由于它有良好的预后，将其与AAH/AIS分为一组。定性变量用χ^2^检验或*Fisher*精确概率法，定量变量用非参数检验*Mann-Whitney U*检验。具有统计学差异的变量进一步进行*Logistic*回归分析。两位影像医师测得的定量变量的观察间一致性由组内相关系数（intraclass correlation coefficient, ICC）分析，ICC < 0.40，表示一致性差; 0.41-0.60，中等; 0.61-0.80，良好; ≥0.81，极好。所有分析在统计软件SPSS 21.0版本中进行。当*P*值小于0.05认为具有统计学意义。

## 2 结果

### 2.1 定量变量观察者间一致性

各个定量变量观察者间一致性极好，除兴趣区CT值SD的一致性较差，其余均大于0.9（表 1）。

**1:** 观察者间一致性 Interobserver consistency

Quantitative variables	ICC	95% CI	
		Lower limit	Upper limit
Long diameter	0.984	0.977	0.990
Short diameter	0.993	0.990	0.995
Height	0.987	0.981	0.992
SD of average CT value of ROI	0.250	-0.122	0.499
Average CT value of ROI	0.992	0.988	0.995
Volume	0.987	0.980	0.991
Average CT value	0.975	0.962	0.983
Volume of solid component	0.984	0.977	0.990
Proportion of solid component	0.964	0.946	0.977
Mass	0.957	0.936	0.972
CT: computed tomography; ROI: Region Interest; ICC: Immunocytochemistry.			

**2:** 定量变量以及单因素分析结果 The quantitative variables and the results of univariate analysis

Quantitative variables	Benign group (*n*=12) (Mean±SD, range)	AAH/AIS/MIA group (*n*=12) (Mean±SD, range)	Invasive adenocarcinoma group (*n*=82) (Mean±SD, range)	*P*1	*P*2
Long diameter (mm)	14.9±5.6 (5.0-22.2)	16.9±5.2 (11.9-27.6)	21.0±6.9 (7.3-45.3)	0.010	0.043
Short diameter (mm)	11.9±4.7 (4.5-19.2)	12.2±3.9 (6.5-22.4)	15.6±5.4 (6.8-33.2)	0.067	0.021
Height (mm)	13.4±5.5 (5.5-24.2)	12.7±5.5 (3.8-21.1)	17.3±7.9 (5.3-44.4)	0.180	0.055
Average CT value (HU) of ROI	-397.0±253.9 (-665.5-54.9)	-377.92±205.3 (-658.7-27.1)	-292.6±173.0 (-781.3-33.3)	0.125	0.129
SD of average CT value of ROI	122.1±57.5 (41.3-220.3)	152.4±50.9 (63.5-222.4)	179.8±119.0 (36.3-1138.1)	0.017	0.353
Volume (mm^3^)	2, 321.1±1, 594.3 (36.3-1, 138.1)	2, 321.1±1, 594.3 (290.5-9, 460.5)	4, 680.1±4, 914.4 (96.0-28, 496.0)	0.268	0.006
Average CT value (HU)	-493.4±93.4 (-685.5-343.0)	-559.8±112.7 (-747.0-330.5)	-460.2±103.3 (-739.0-257.5)	0.464	0.005
Mass (g)	1.4±1.0 (0.2-2.8)	1.2±2.1 (0.2-7.6)	3.1±3.7(0.02-21.9)	0.317	0.003
Volume of solid component (mm^3^)	1, 173.3±923.0 (0-2474)	800.5±1, 969.7 (0-6, 693)	2, 764.6±3, 521.4 (0-21, 226)	0.414	0.001
Proportion of solid component (%)	47.7±20.8 (0-66.5)	26.4±26.1 (0-70.8)	49.4±22.9 (0-81.4)	0.844	0.005
*P*1: benign group *vs* malignant group (AAH/AIS/MIA group+invasive adenocarcinoma group); *P*2: AAH/AIS/MIA group *vs* invasive adenocarcinoma group. AAH: atypical adenomatous hyperplasia; AIS: adenocarcinoma *in situ*; MIA: minimally invasive adenocarcinoma.					

**1:**
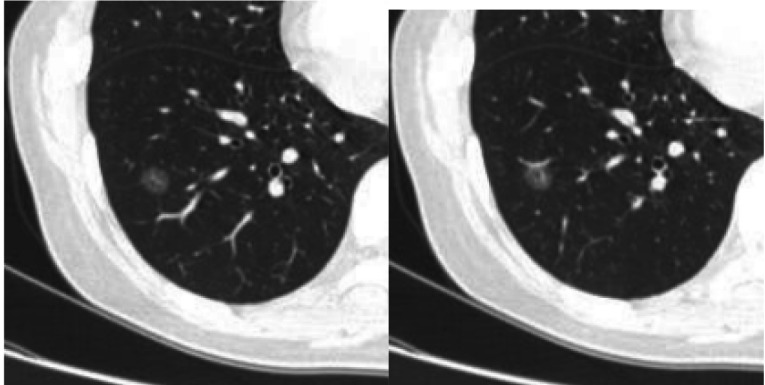
女性，45岁，右肺下叶磨玻璃密度结节影，球形、边缘光滑、瘤肺界面清晰，病理结果：AIS。 Female, 45 years old, a ground-glass nodule in right lower lobe, spherical, smooth margin, clear tumor-lung interface, pathology: AIS.

**2:**
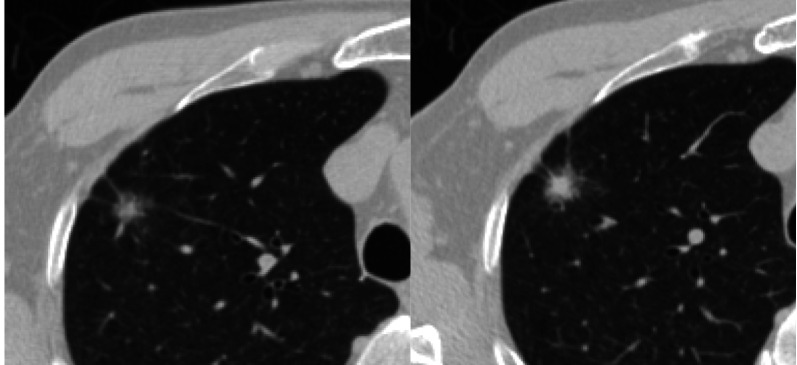
男性，62岁，右肺上叶亚实性结节，球形、边缘毛刺、胸膜牵拉征、瘤肺界面清晰，病理结果：侵袭性腺癌。 Male, 62 years old, a part-solid nodule in right upper lobe, spherical, spiculated margin, pleural retraction, clear tumor-lung interface, pathology: invasive adenocarcinoma.

**3:**
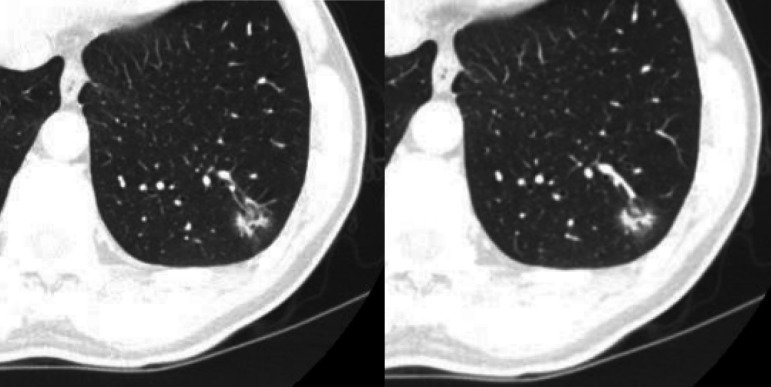
男性，45岁，左肺下叶亚实性结节，球形、瘤肺界面不清晰，病理结果：炎性假瘤。 Male, 45 years old, a part-solid nodule in left lower lobe, spherical, unclear tumor-lung interface, pathology: inflammatory pseudotumor.

**3:** 定性变量以及单因素分析结果 The qualitative variables and the results of univariate analysis

Qualitative variables		Benign group	AAH/AIS/MIA group	Invasive adenocarcinoma group	*P*1	*P*2
Nodule type	Ground-glass	4	4	9	0.083	0.036
	Part-solid	8	8	73		
Tumor-lung interface	Clear	7	11	79	0.000	0.454
	No clear	5	1	3		
Lobulation	Yes	2	6	62	0.000	0.064
	No	10	6	20		
Pleural traction	Yes	2	4	56	0.002	0.019
	No	10	8	26		
Air bronchus sign	Yes	2	4	45	0.010	0.442
	No	10	8	37		
Pulmonary vascular abnormality	Yes	7	9	71	0.023	0.292
	No	5	3	11		
*P*1: benign group *vs* malignant group (AAH/AIS/MIA group+invasive adenocarcinoma group); *P*2: AAH/AIS/MIA group *vs* invasive adenocarcinoma group.						

**4:** 良恶性结节二元Logistic回归分析结果 The binary logistic regression analysis of benign and malignant group

Item	OR	95%CI	*P*
Tumor-lung interface	38.1	5.0-287.7	0.000
Lobulation	1		0.074
Pleural traction	1		0.105
Air bronchus sign	7.9	1.3-49.3	0.027
Pulmonary vascular abnormality	7.2	1.4-37.0	0.019
Long diameter	1		0.904
SD of average CT value of ROI	1		0.586

**5:** AAH/AIS/MIA组与侵袭性腺癌组二元Logistic回归分析结果 The binary logistic regression analysis of AAH/AIS/MIA group and invasive adenocarcinoma group

Item	OR	95%CI	*P*
Nodule type	1		0.754
Pleural traction	1		0.540
Long diameter	1		0.319
Short diameter	1		0.215
Volume	1		0.283
Average CT value	1		0.397
Mass	1		0.452
Volume of solid component	1		0.338
Proportion of solid component	1.04	1.01-1.06	0.006

### 2.2 良恶性结节鉴别单因素分析

定性变量中，瘤肺界面（*P*=0.000）、分叶状（*P*=0.000）、胸膜牵拉征（*P*=0.002）、空气支气管征（*P*=0.010）以及肺血管异常（*P*=0.023）在良性结节与恶性结节间存在统计学差异。定量变量中仅结节长径（*P*=0.010）、结节兴趣区CT值的标准差（*P*=0.017）在良恶性结节间存在统计学差异，纳入*Logistic*回归分析。

### 2.3 腺癌恶性侵袭度单因素分析

定性变量中结节类型（*P*=0.036）、胸膜牵拉征（*P*=0.019）在AAH/AIS/MIA组与侵袭性腺癌组结节之间存在统计学差异。定量变量中结节长径（*P*=0.043）、结节短径（*P*=0.021）、结节体积（*P*=0.006）、结节平均CT值（*P*=0.006）、结节的实性成分体积（*P*=0.001）、结节的实性成分所占比例（*P*=0.005）、结节质量（*P*=0.003）在AAH/AIS/MIA组与侵袭性腺癌组结节之间存在统计学差异，纳入*Logistic*回归分析。

### 2.4 多因素*Logistic*回归分析

良性组与恶性组分析结果显示清晰的瘤肺界面、空气支气管征以及肺血管异常的出现是恶性结节的重要指标。清晰的瘤肺界面、空气支气管征以及结节内血管异常是恶性结节的重要预测指标，风险比分别为38.1（95%CI: 5.0-287.7; *P* < 0.01）、7.9（95%CI: 1.3-49.3; *P*=0.03）、7.2（95%CI: 1.4-37.0; *P*=0.02）。AAH/AIS/MIA组与侵袭性腺癌组结果显示仅更高的实性成分所占比例是侵袭性腺癌与AAH/AIS/MIA组鉴别的重要指标，其风险比分别为1.04（95%CI: 1.01-1.06, *P*=0.01）。

## 3 讨论

亚实性结节术前CT预测腺癌病理类型对病人管理具有重要意义。最近研究对于预后较好的病灶（即AIS、MIA），肺段切除的癌症相关生存率与肺叶切除相当，并且有更少的并发症^[4, 5]^。肺段切除可保留更多有功能的肺实质，对于年龄较大或双侧有多个结节的患者尤为重要^[6]^。

本研究表明清晰的瘤肺界面是恶性结节的重要预测指标，风险比为38.1。Fan^[7]^、Nambu^[8]^也指出，清晰的瘤肺界面是亚实性结节良恶性的鉴别点。在本研究中，除了4个瘤肺界面不清晰的病例外，所有恶性病例均出现清晰界面（恶性结节组，瘤肺界面90例清晰，4例不清晰）。而我们的良性结节组12个结节中5个瘤界面不清晰。恶性病变的瘤肺界面，病理上主要为肿瘤浸润性生长所致，通常界面清晰。相反，良性病变由于炎性细胞的浸润，瘤肺界面通常是不清晰^[7]^。

空气支气管征是我们发现的另一具有重要预测价值的恶性结节征象，风险比为7.9。空气支气管征病理基础为是肿瘤细胞沿着肺泡壁以附壁方式生长，造成肺泡腔内细胞堆积，表现为影像中的磨玻璃密度影，其早期不侵犯细小支气管，使其在结节内显影。当肿瘤继续增长时，其内可出现纤维组织，纤维组织收缩会使细小支气管扭曲或扩张，含气的支气管影可能会明显。既往的研究也表明空气支气管征是腺癌的预测指标^[8, 9]^。

结节内血管异常对预测恶性结节也具有重要价值，风险比为7.2。结节内血管异常可能是由于肿瘤内的纤维组织增生反应的收缩引起肿瘤内血管扩张，僵直或扭曲^[10]^。

实性成分所占比例在AAH/AIS/MIA组与侵袭性腺癌组之间存在统计学差异，侵袭腺癌组更易表现更大的实性成分所占百分比。已知CT上结节的实性成分可能与纤维灶、肺泡坍塌或肿瘤增生有关。之前的几项研究表明对于部分实性结节，实性成分的大小与病灶的恶性度有关，实性成分越大恶性、侵袭性的可能性越大，并且实性成分大于50%预示着侵袭性病变可能性大^[11-16]^。

本研究的局限性：首先本研究只选取了结节手术切除的病人，有一定的选择偏倚，恶性可能性更高。另外我们的良性组、侵袭前组+MIA组的样本量相对较小，对结论会有一定影响。

## References

[1] Austin JH, Müller NL, Friedman PJ (1996). Glossary of terms for CT of the lungs: recommendations of the Nomenclature Committee of the Fleischner Society. Radiology.

[2] Benveniste AP, Godoy MC, Truong MT (2015). Case of the season: management of the subsolid pulmonary nodule. Semin Roentgenol.

[3] Truong MT, Ko JP, Rossi SE (2014). Update in the evaluation of the solitary pulmonary nodule. Radiographics.

[4] Okami J, Ito Y, Higashiyama M (2010). Sublobar resection provides an equivalent survival after lobectomy in elderly patients with early lung cancer. Ann Thorac Surg.

[5] Scott WJ, Howington J, Feigenberg S (2007). Treatment of non-small cell lung cancer stage Ⅰ and stage Ⅱ: ACCP evidence-based clinical practice guidelines (2^nd^ edition). Chest.

[6] Cohen JG, Reymond E, Lederlin M (2015). Differentiating pre- and minimally invasive from invasive adenocarcinoma using CT-features in persistent pulmonary part-solid nodules in Caucasian patients. Eur J of Radiol.

[7] Fan L, Liu SY, Li QC (2012). Multidetector CT features of pulmonary focal ground-glass opacity: differences between benign and malignant. Br J Radiol.

[8] Nambu A, Araki T, Taguchi Y (2005). Focal area of ground-glass opacity and ground-glass opacity predominance on thin-section CT: discrimination between neoplastic and non-neoplastic lesions. Clin Radiol.

[9] Hu H, Wang Q, Tang H (2016). Multi‐slice computed tomography characteristics of solitary pulmonary ground‐glass nodules: Differences between malignant and benign. Thorac Cancer.

[10] Zhang Y, Qiang JW, Ye JD (2014). High resolution CT in differentiating minimally invasive component in early lung adenocarcinoma. Lung Cancer.

[11] Kodama K, Higashiyama M, Yokouchi H (2002). Natural history of pure ground-glass opacity after long-term follow-up of more than 2 years. Ann Thorac Surg.

[12] Mun M, Kohno T (2007). Efficacy of thoracoscopic resection for multifocal bronchioloalveolar carcinoma showing pure ground-glass opacities of 20 mm or less in diameter. J Thorac Cardiovasc Surg.

[13] Ohtsuka T (2006). A clinicopathological study of resected pulmonary nodules with focal pure ground-glass opacity. Eur J Cardiothorac Surg.

[14] Kodama K, Higashiyama M, Takami K (2008). Treatment strategy for patients with small peripheral lung lesion(s): intermediate-term results of prospective study. Eur J Cardiothorac Surg.

[15] Kim TJ, Goo JM, Lee KW (2009). Clinical, pathological and thin-section CT features of persistent multiple ground-glass opacity nodules: comparison with solitary ground-glass opacity nodule. Lung Cancer.

[16] Park CM, Goo JM, Kim TJ (2008). Pulmonary nodular ground-glass opacities in patients with extrapulmonary cancers: what is their clinical significance and how can we determine whether they are malignant or benign lesions?. Chest.

